# Current Classification of Seizures and Epilepsies: Scope, Limitations and Recommendations for Future Action

**DOI:** 10.7759/cureus.10549

**Published:** 2020-09-20

**Authors:** Shah T Sarmast, Abba Musa Abdullahi, Nusrat Jahan

**Affiliations:** 1 Neurology, California Institute of Behavioral Neuroscience & Psychology, Fairfield, USA; 2 Neurology/Neuroscience, California Institute of Behavioral Neuroscience & Psychology, Fairfield, USA; 3 Internal Medicine, California Institute of Behavioral Neuroscience & Psychology, Fairfield, USA

**Keywords:** seizure, international league against epilepsy (ilae), objectives, scope, epilepsy, classification

## Abstract

The classification of seizures and epilepsies by the International League Against Epilepsy (ILAE), 2017 is the most recent classification model which aimed to simplify terminologies that patients and their caregivers can easily understand, identify seizures that have both focal and generalized onset and incorporate missing seizures. We have exhaustively reviewed the studies, discussed its scope, outlined its limitations and gave recommendations that could help in forming subsequent reviews. We have also described the terminologies that have been replaced, redefined or removed to have a clear view of the previous and the current classification models. We have recommended the use of multidimensional classification model which incorporated the clinical semiology, disease location, etiology and associated comorbidities. The benefits of this model is for prompt diagnosis which will results into early management and then better patient outcomes. It would also have a profound effects on the kind of treatment patients might receive especially in developing countries where there are scarcity of the diagnostic techniques. Overall, in this study we have reviewed the current study on seizures and epilepsy classification model by ILAE, 2017 to clarify the descriptions and coverage, outlined some limitations and suggested recommendations.

## Introduction and background

The most recent classification of seizures and epilepsies was the International League Against Epilepsy (ILAE), 2017, which was published in March 2017. This new classification is better organized with a clear elucidation of terminologies and list some new seizure types. Better diagnosis and management of seizures and epilepsy is achieved when classified and grouped into similar entities as different drugs are usually effective for different seizure types [1]. In this current classification by ILAE, the clinical features of epilepsy are categorized into three levels: the seizures, the epilepsies, and the epilepsy syndromes. Emphases have been made to consider etiology and comorbidities at each level[2]. Also, epilepsy is declared a curable disease rather than a disorder. It is said to be resolved after ten years of the seizure-free period with the last five years spent without medications, or the patient is no longer at risk for age-related epilepsy syndrome [3].

Seizures are defined as transient symptoms and signs due to abnormal excessive or simultaneous neuronal activity of a population of neuronal cells in the brain. While epilepsy is defined as a chronic disorder of the brain characterized by an enduring disposition towards recurrent unprovoked seizures and by the neurobiological, cognitive, psychological, and social consequences of this condition. The diagnosis of epilepsy requires at least two unprovoked seizures occurring greater than twenty-four hours apart. A syndrome is defined as a characteristic seizure associated with abnormal investigations that occur together in a recognizable pattern. It usually includes more than one type of epilepsy[4]. The possibility of having a recurrent seizure can be suspected when the patient's electroencephalogram (EEG) shows an epileptiform activity or when epileptogenic abnormality on brain imaging was detected[5]. Several factors are considered during the classification of epilepsy, and these include mode and age of onset, seizure pattern, family history, EEG, and MRI findings [6].

However, these definitions have some drawbacks as a person may be at a very high risk of having second seizure even after the first one and, therefore, should be considered to have epilepsy. Also, some benign epilepsy syndromes like benign Rolandic epilepsy may be identified after one seizure with EEG as centrotemporal spikes. It is interesting to know that all seizures are not unprovoked, some occurs after provocation as in some reflex seizures like photosensitive epilepsy. Therefore, the definition of epilepsy has been revised and expanded from having two or more unprovoked seizures more than twenty-four hours apart to including those with one seizure and a high likelihood (more than 60%) of having another [3]. More than 60% was taken because it is the lower limit of the confidence interval for the individual with two unprovoked seizures having another seizure [5]. Many health professionals other than neurologists or those with specialized training in epilepsy might find it challenging to comprehend the classification and terminologies used in seizures and epilepsies. Also, many specialists have suggested other forms of classification different from that of ILAE, which might be useful in some settings. Therefore, we have extensively reviewed the published literature of the current classification model by the ILAE, 2017 to elucidate on the terminologies, highlight some limitations and propose some recommendations.

## Review

Scope

Objectives

This is the first official classification of a seizure by the ILAE since 1989. The objectives of the revision are to use terminologies that are more understandable by both clinicians and patients, recognize seizures that have both focal and generalized onset and including some missing seizures [4].

Benefits

The benefits of the classification are enormous to both the patient and the clinician. It categorizes seizures into individual forms, with each having specific characteristics, onset, and clinical presentation with consequent attainment of better patient care. As different seizure types have different medications specific for each type, this classification could help to identify a particular drug that is effective to a specific type of seizure. It also helps in identifying comorbidities and prognosis associated with a specific type of seizure [7]. The classification can be applied for both adult and pediatric seizures as well as epilepsies, except for neonatal seizures, classified separately [5].

Another advantage of classification includes merging both seizures and epilepsies in a single format, and the ability to classify seizures based on the available investigations in both resource-rich and resource-poor settings as seizures are classified here solely on clinical presentation [8]. Pharmacologically, this classification has great relevance as focal seizures, regardless of the part of the brain involved, were shown to respond to a specific set of anticonvulsants. In contrast, drugs for generalized seizures depend on its specific type [9].

Newer Terminologies

The older terminologies like convulsion, dyscognitive, simple-partial, complex-partial, psychic, and secondarily generalized were removed from the new classification due to serious criticism, as they were not completely understood by the patient or the public since these were the ones that watch the seizure event, not the physician in most of the cases [4]. Also, most of the older terminologies are not descriptive of the real seizure event; for example, the term partial means part of a seizure rather than anatomic location. Therefore, it is replaced with a focal, which describes the locus of the seizure event localized to one cerebral hemisphere.

In the same way, the term focal seizure with secondary generalization was replaced by focal to bilateral tonic-clonic terminology as it better describes the propagation pattern of the seizure [1]. Similarly, simple and complex terms are confusing to patients as simple might mean not serious to him, and complex might be taken as the only severe or difficult seizure. Therefore, simple partial was replaced by focal aware; complex partial. Psychomotor and dyscognitive were replaced by a single term, focal impaired awareness. Grand mal was replaced by generalized tonic-clonic, focal to bilateral tonic-clonic, and unknown onset tonic-clonic; and infantile spasm replaced by epileptic spasm [1,10].

Other newer terminologies include emotional seizure, cognitive seizure, absence with eyelid myoclonia, myoclonic, atonic, focal myoclonic, focal tonic, focal epileptic spasms, behavior arrest, unaware, and unclassified seizures. The term unconsciousness is avoided in this revised classification as it could be confusing as to whether the patient has really lost consciousness or is just not aware of what has happened around him during the event. Therefore, a new term aware was introduced because a person may be fully conscious but not aware of what happens around him [2]. Table 1 below shows the comparison between old and new terminologies.

**Table 1:** Comparison Between Old and New Terminologies

Old Terminology	New Terminology
Partial Seizure	Focal Seizure
Focal seizure with secondary generalization	Focal to bilateral tonic-clonic
Simple Partial	Focal Aware
Psychomotorand Dyscognitive	Focal impaired awareness
Grand mal	Generalized tonic-clonic
Infantile Spasm	Epileptic Spasm

New Seizures

The new seizure types included in this classification are: (i) focal motor: epileptic spasms, hyperkinetic and automatism - in this new classification, seizures like myoclonic, tonic, tonic-clonic, clonic and atonic which were considered only generalized are now included in focal onset seizures; (ii) focal non-motor: emotional or behavioral arrest; (iii) generalized: epileptic spasms, myoclonic-atonic, myoclonic-tonic-clonic, and absence with eyelid myoclonia​ [11].

Framework of the Classification

Broadly, four components are included in the new classification

1. Seizures (focal onset, generalized onset, unknown onset);

2. Epilepsies (focal, generalized, combined generalized, and focal, unknown);

3. Epilepsy syndromes; and

4. Etiology (structural, genetic, metabolic, infectious, immune, unknown).

The new classification also encourages patient characterization based on the available resources like electroencephalography (EEG) findings, video recording, neuroimaging, or genetic workup [1]. During this revised publication, three studies were published in which two of the studies [7,11] highlighted the revised classification and the third study [10] was just a guide on how to use the revised terminology in the clinical practice [4].

A study done by Scheffer and colleagues [7] extensively discussed the revised classification of epilepsy and epilepsy syndromes, emphasizing etiology, and associated comorbidities from the point of presentation to the final stage of patient management. Although it is mainly for epilepsy, the classification begins with classifying seizures because epilepsy is a progression of seizures [4].

A study done by Fisher et al.is a discussion on the revised classification of seizures, in which new seizure types were introduced, and older terminologies were replaced with newer ones [11]. In this study, two categories of seizure classification have been made: basic and expanded classification. The basic classification is for general consumption by both health care professionals and the general public. In contrast, the expanded classification is meant for specialists such as neurologists, psychiatrists, neurosurgeons, or those with special expertise in epilepsy [5]. 

Seizure Classification and Definition of Terms

In this revised classification, seizures are classified based on three features:

1. Origin of the seizure in the brain.

2. Degree of awareness during the seizure.

3. Level of body movement.

Based on the first feature, a seizure can be focal onset or generalized onset. The second feature implies either awareness (knowledge of self and environment) intact or awareness impaired while the last feature means that seizure can be motor or non-motor onset. Therefore, seizures are classified into three:

1) Focal onset seizures, which are subsided into three subtypes:

a) Retained awareness/impaired awareness

b) Motor/non-motor onset

c) Focal to bilateral tonic-clonic.

2) Generalized onset seizures, which consist of motor or non-motor (absence) onset seizures.

3) Unknown onset seizures can be motor/non-motor or unclassified [12]. However, classification based on awareness is fully optional and can be omitted, especially when it is difficult to establish the level of awareness impairment as in sudden, brief seizures [11]. 

The focal seizure may occur while the patient is fully aware of the events occurring around him during the seizure; this called focal aware seizures. However, in the majority of the instances, the patient may lose awareness of some events during the seizure, and this is called focal impaired awareness. There may be associated loss of memory (amnesia) during the seizure event. The term aura is commonly associated with focal seizures and can occur alone like an aura of deja vu, a strange taste or smell, or a rising sensation in the stomach, lip-smacking, and hand rubbing. In this case, the aura is referred to as a focal aware seizure [4]. Focal seizures are named based on first signs or symptoms that appear at the beginning of the seizure, even if the later signs or symptoms are more remarkable than the first one. The only exception is impairment of awareness, which always determines the type of the seizure even if the impairment occurs at the last phase of the seizure event. This is called the Rule of First, and clinically the first symptoms, although argued, determine the locus of the seizure [3,5]. 

**Figure 1:**
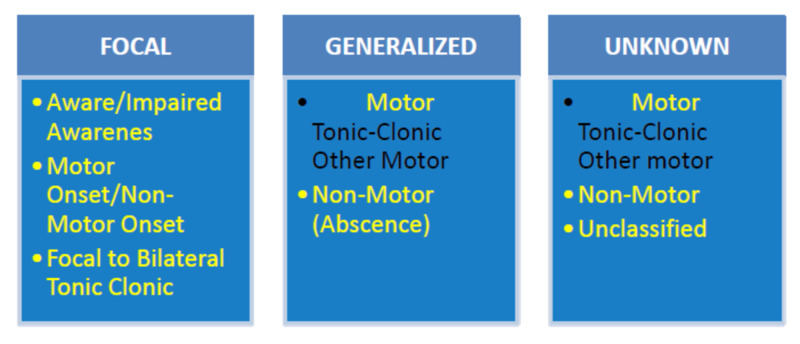
ILAE 2017 Classification of Seizure Types: Basic Version ILAE: International League Against Epilepsy

**Figure 2:**
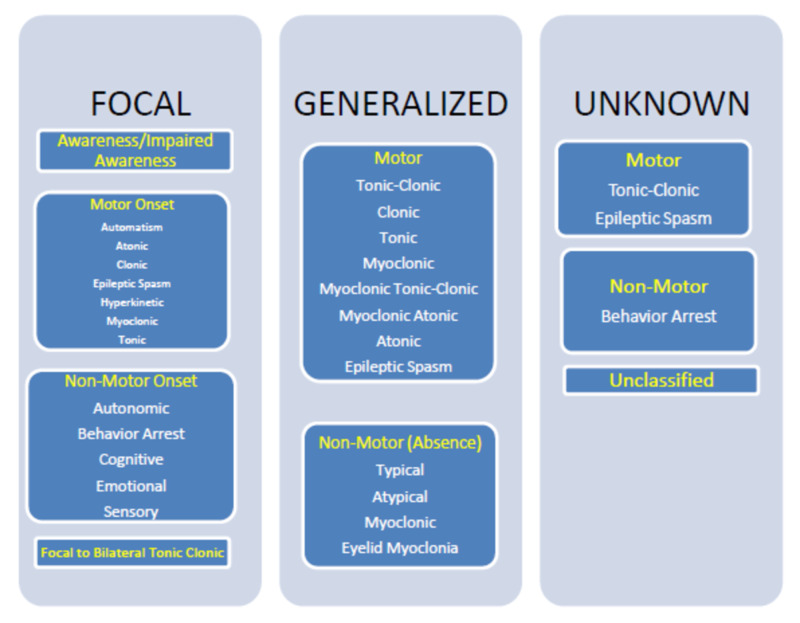
ILAE 2017 Classification of Seizure Types: Expanded Version ILAE: International League Against Epilepsy

Focal seizures occur when abnormal electrical activities originate from one side of the cerebral hemisphere, which can progress to the other side. Awareness varies in focal seizure and can be present or absent. There may be associated jerking of a single limb (arm or leg), which can progress to involve both sides of the limbs (both arms and legs), where it is referred to as a focal to a bilateral tonic-clonic seizure. Generalized seizures mean that abnormal electrical activities started simultaneously from both rights and left cerebral hemispheres and then spread to the other brain neuronal networks evidenced by the patient clinical presentation or EEG features [4]. Figures 1-2 below are the basic and expanded versions of the classification respectively.

A) Focal motor (associated muscles movement) seizure [3,4,10,11]. Table 2 below shows the types of focal motor seizures.

**Table 2:** Focal Motor Seizures

Seizure Types	Meaning
1. Focal Automatism Seizure	Commonly misdiagnosed and disregarded as a seizure. It is repeated motor activity that usually occurs under impaired awareness and sometimes followed by amnesia and characterized by repeated action, such as saying something repeatedly, lip-smacking, rubbing, or wandering. It is a common feature of focal impaired awareness seizure.
2. Focal Atonic Seizures	Seizures with loss of muscle tone that occurs suddenly and lasts for a few seconds,It can occur on one side of the body or in One limb. Awareness is usually retained.
3. Focal Tonic Seizures	Seizures with a sustained increase in muscle contraction that lasts for a few seconds or minutes and present clinically as stiffening of a limb or the neck.
4. Focal Clonic Seizures	Sustained rhythmical jerking of a group of muscles that occur either symmetrically or asymmetrically.
5. Focal Epileptic Spasms	Sudden uncontrolled and sometimes painful muscle contractions that commonly occur in children. Clinically present as sudden flexion of the waist and flexion or extension of the arms and legs, which may occur in clusters and can be focal, generalized, or of unknown onset. It is usually diagnosed by video-EEG. When it occurs in infants, it is called Infantile Spasm. Awareness is usually retained.
6. Focal Hyperkinetic Seizures	Seizures associated with an exaggerated, and often uncontrolled muscles activities such as agitated kicking, thrashing, and peddling during the seizure.
7. Focal Myoclonic Seizures	It may be similar to clonic seizures but are usually brief, unsustained muscle contractions that occur suddenly and last for few seconds or even less than a second or just an irregular jerking in one part of the face or body. Awareness is usually retained.

B) Focal non-motor (not associated with muscles movement) seizure [3,4,10,11]. Table 3 below shows the types of focal nonmotor seizures.

**Table 3:** Focal Non-motor Seizures

Seizure Types	Meaning
1. Focal Non-motor Autonomic Seizures	These are seizures affecting autonomic nervous system presenting with symptoms like rising sensation in the stomach, hot and cold feelings, a strange taste or smell, etc
2. Focal Non-motor Behavior Arrest Seizures	These are seizures presenting with the cessation of all activities and unresponsiveness for the entire duration of the seizure event.
3. Focal Non-motor Cognitive Seizures	When there are hallucinations, illusions, deja vu, or impaired speech during the seizure event, the patient is said to have had cognitive seizures.
4. Focal Non-motor Emotional Seizures	These seizures refer to non-motor seizures that begin with panic, anxiety, fear, joy, crying, depression, or any other emotion.
5. Focal Non-motor Sensory Seizures	These seizures present with abnormal sensations such as visual, olfactory, auditory, gustatory, somatic hallucination, or vertigo?

Generalized seizures can be motor or non-motor. The motor seizures include tonic-clonic, clonic, tonic, myoclonic, myoclonic-tonic-clonic, myoclonic-atonic, atonic, or epileptic spasms, whereas the non-motor are either typical or atypical absence seizures or seizures with myoclonic activity or eyelid myoclonia [3.4]. Most of the generalized seizures are associated with impairment of awareness, and therefore, the classification based on awareness is voluntarily omitted [6]. 

A) Generalized motor seizure [3,4,10,11]. Table 4 below shows the types of generalized motor seizures.

**Table 4:** Generalized Motor Seizure

Seizure Types	Meaning
1. Myoclonic-tonic-clonic seizures	This seizure is usually observed in individuals with juvenile myoclonic epilepsy. It is characterized by arms jerking, then tonic stiffening, and lastly, clonic rhythmical jerking.
2. Myoclonic-atonic seizures	This seizure is commonly seen in patients with Doose syndrome and was previously referred to as myoclonic Astatic seizures. It is characterized by brief jerking of limbs or trunk, followed by a limp drop.

B) Generalized non-motor seizures [3,4,10,11]. Table 5 below shows types of generalized nonmotor seizure.

**Table 5:** Generalized Non-motor Seizures EEG: electroencephalogram

Seizure Types	Meaning
1. Typical Absence Seizures	These seizures are characterized by interruption of activities that occur suddenly with a blank stare and occasionally associated with deviation of the eyes that lasts for a few seconds to half a minute with subsequent rapid recovery. May be associated with flicking of eyelids.
2. Atypical Absence Seizures	It has a slow onset with significant changes in muscle tone that is more pronounced than in typical absence. It has a slow spike-waves at EEG, usually less than three per second.
3. A myoclonic-absence seizure	This seizure starts with few rhythmical jerks, which is then followed by a staring spell.
4. Eyelid myoclonia	In this seizure, there is sudden forceful upward jerking of eyelids, which may be associated with the staring spell (absence seizure). It usually stimulated by the closure of the eyes.

Seizures of unknown onset are seizures that occur either in sleep or in a condition that cannot be described as the patient is alone or the witness cannot describe it. Also, when the clinician is certain that the information given is a seizure event but cannot describe it due to incomplete information, it is regarded as an unclassified seizure. Therefore, the term unknown onset is just like a nickname but not the characteristic of the seizure [1].

Epilepsy Classification and Definition of Terms

Epilepsy is classified into four main types in this revised classification: 1) focal; 2) generalized; 3) combined generalized and focal; 4) unknown.

The group of combined generalized and focal epilepsy is a new terminology introduced in the type of epilepsy. Also, Idiopathic generalized epilepsy has been replaced by genetic generalized epilepsy, which comprises of childhood absence epilepsy, juvenile absence epilepsy, juvenile myoclonic epilepsy, and generalized tonic-clonic seizures alone. Moreover, terms like epileptic encephalopathy and developmental encephalopathy have been renamed [1,12].

In this revised classification, epilepsy is classified into three levels: the seizure type, epilepsy, and epilepsy syndrome. The diagnosis of epilepsy always starts with determining the type of seizure first, then followed by the type of epilepsy. Based on further clinical evidence and the available investigation findings, epilepsy could then be diagnosed as epilepsy syndrome [7]. This form of classification was designed to cater to classifying epilepsy in different clinical environments. That is in a setting where patients present with seizures that do not fit the definition of epilepsy, and no electroencephalography, a diagnosis of epileptic seizure will thus be made. Also, in a setting where patients present with clinical details that fit the definition of epilepsy but no electroencephalography (EEG), video electroencephalogram (VEEG), computed tomographies (CTs), magnetic resonance imaging (MRI) to further categorize the seizure as either epilepsy or epilepsy syndrome, then a diagnosis of epilepsy should be made. However, when investigations are feasible, a diagnosis at all three levels should be sought as well as the etiology of the individual's epilepsy [7].

**Figure 3:**
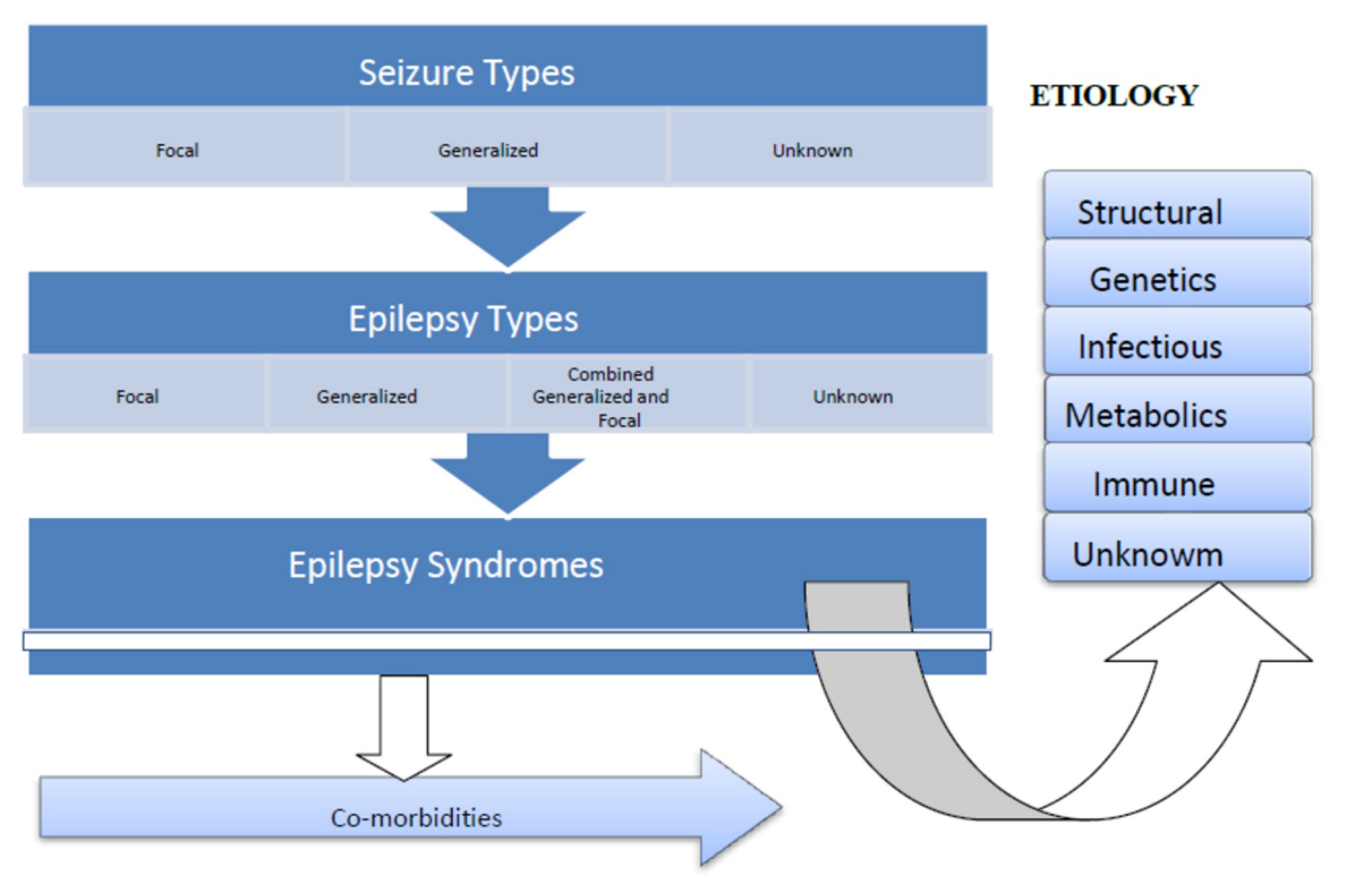
ILAE 2017 Classification of Epilepsies ILAE: International League Against Epilepsy

Focal epilepsy is a continuum of seizures, unifocal, and multifocal disorders that are confined to one hemisphere and can present with any type of focal seizure. It is diagnosed clinically but supported by an interictal EEG with focal epileptiform discharges. Similarly, generalized epilepsy consists of many seizure types, and a patient can present with any type of generalized seizure, either motor or non-motor. Diagnosis is based on generalized spike-wave activity on EEG, and more importantly, clinical presentation [7]. Combined generalized and focal is a condition in which a patient has both focal and generalized seizures, which is usually diagnosed by VEEG and commonly occurs in infants or children with severe epilepsies. In a situation where the clinician cannot categorize seizures as either for focal or generalized, especially in a resource limiting setting, the seizure is therefore referred to as unknown [4]. Figure 3 below shows types of epilepsy.

Instruction Manual

Another distinguishing feature of this current classification is the formation of the instruction manual, which serves as an introduction to the classification and guidance on how to use the current classification. The manual summarizes the scope used in the classification by the use of tables, and glossary was made for the relevant terminologies. Old and new terminologies were compared, and numerous examples of clinical scenarios were provided for easy comprehension. A general overview of both basic and expanded classification was provided, and definitions of the new terminologies were elucidated. Common descriptors for the clinical features of seizures were included, and guidelines or rules to be used clinically while classifying seizures were summarized in the manual [10]. 

Limitations of the 2017 ILAE classification

Some specialists have criticized the ILAE classification due to a number of limitations contained therein. For example, the classification does not take into cognizance the pathology and pathophysiologic mechanisms of seizures and epilepsy [12]. We, therefore, identified the following drawbacks: 

Subjectivity of Some of the New Terms

By subjectivity, we mean the ability of the terms to be understood wrongly or differently by different people, and therefore cause confusion. Some of the terms used like "awareness" were highly subjective in definition, and some patients might perceive the term as synonymous with non-serious seizure. Also, awareness could, sometimes, not be described by patients. For example, in the "focal aware sensory seizure," the sensory auras could not be explained by some patients as they occur spontaneously in some patients [13]. Additionally, the term awareness, which invariably related to the conscious level of a patient, is often not focal as multiple cortical and subcortical structures were shown to be affected. Similarly, the term behavior arrest is subjective and could be confused with absence seizure, especially atypical absence seizure. Furthermore, the dichotomy between "focal impaired awareness seizure" and "focal cognitive seizure" might be confusing. As many studies have shown that seizures with overall impairment of awareness/consciousness generally have impaired cognitive function, therefore separating the seizures, which obviously could be considered as one, is a tautology [14]. Also, a form of classification may be assigned to a particular seizure condition, which, upon further investigation, could be changed to a different form of classification. This may have a negative effect on patient management as the specific drug may be used for specific seizure types [13]. 

Concept of Consciousness

Consciousness has been voluntarily removed from this classification in an attempt to use simpler terminologies. Instead, the term awareness was used [10]. However, the term is still valid and clinically significant as many seizures present with an impaired conscious level, which could indicate the severity of the patients' condition upon which safety and legal issues could be built, the concept that awareness cannot provide [15]. Further stressing the importance of consciousness is the result of a survey conducted by Mathern et al. through Epilepsia, in which about 77% of the respondent believe that term consciousness was significant and therefore should be maintained in seizure and epilepsy classification [16]. Additionally, patients with sensory-motor deficits and amnesic may have behavioral unresponsiveness, which may present as impaired awareness, but consciousness may not be affected [17]. 

Poor Incorporation of Clinical Features

Although claimed by ILAE that semiologic features were used in making the classification [10], but other critical clinical features like preictal and postictal features were not included as only ictal features were considered. This is crucial as symptomatology evolved from the preictal to the ictal and postictal features. Therefore, terms like "focal tonic with somatosensory auras”, ‟generalized tonic-clonic with the cognitive prodrome”, ‟generalized atonic with postictal sleep” should have been used to incorporate full semilogic features [13]. This evolution of seizure symptomatology would serve as an important tool in identifying the part of the cortex involved as either frontal, temporal, parietal, or occipital. For instance, temporal lobe seizures commonly preceded with epigastric auras; frontal lobe seizures are preceded with somatosensory auras localized to the chest or epigastrium, with olfactory auras suggesting orbitofrontal lobe involvement [18]. Similarly, parietal lobe seizures present with somatosensory auras but of numbness, painful or tingling sensations, whereas occipital lobe seizures are commonly preceded with visual auras [19]. Another advantage of incorporating this evolution into seizure classification is the ability of both patients and clinicians to recognize seizure events early, which would facilitate pre-emptive intervention and thus better quality of life [20]. 

Use of Confusing Terminologies

Although clinical features could give a clue about the seizure onset, the definitive mode of onset can only be identified from the diagnostic apparatus such as EEG, CTs, or MRI as it indicates the abnormal electrical activities of a particular brain location [21]. Furthermore, designating a seizure as either focal or generalized is synonymous to the pathophysiologic mechanism of the seizure [22]. Therefore, both the epileptogenic zone and the pathophysiology, in addition to the semiology, are needed in classifying seizure as either focal or generalized. Additionally, seizures that do not have motor activity could barely be classified as either focal or generalized. For example, abnormal sensation could be due to either localized or generalized abnormal electrical activity of the brain, which can only be determined from EEG or other diagnostic techniques. Even some motor seizures like tonic seizures involving limb bilaterally can result from either the localized motor area or diffusely from both the cerebral cortex and only EEG can differentiate [23]. 

Undefined Roles of Etiology and Comorbidity

Although, the emphasis has been made on identifying the etiology and comorbidities [7], no clear criteria or guidelines on the role of comorbidities and etiological factors in classifying seizures and epilepsies. The terms used are only descriptive of the clinical manifestation of an epileptic event. For example, a patient may have a bilateral tonic-clonic seizure, and the MRI may show a large frontotemporal tumor or patient with generalized tonic-clonic seizure and may have background comorbidity of depression. With ILAE classification, the etiology (frontotemporal tumor) and the comorbidity (depression) are not included [13]. Etiological factors and comorbidities such as anxiety, depression, or dementia present a high burden among epileptic patients. Therefore, guidelines for early detection and prompt treatment are of paramount importance [24], which are unfortunately lacking in this current classification. Formulating rules in investigating comorbidities would greatly improve the current understanding of the pathophysiology of epilepsy and could stimulate further researches, especially along with genetic studies [25]. 

Missing Definition of Some Important Terminologies

Descriptors and identifiers regarding provoked and unprovoked seizures were completely missing or poorly defined in the classification, although the concept of etiology has been highlighted in epilepsy classification [7], which could give an idea on provoked and unprovoked. Seizures that occur following acute brain insults are referred to as provoked and can be caused by many triggers, including central nervous system (CNS) infections, cerebrovascular diseases, head injury, metabolic derangement, or iatrogenic from the neurosurgical intervention [26], whereas unprovoked seizures occur spontaneously. The benefit of stressing this is that it would encourage proper evaluation of patients with basic and less expensive investigations such as complete blood count, electrolytes, urea and creatinine, and blood glucose level [27]. It could also help in preventing the overzealous use of antiepileptic drugs and timely identification of predictors of both provoked and unprovoked[28]. 

Recommendations

Multidimentional Classification

Definitive characterization of a seizure or epilepsy requires the incorporation of many dimensions involving the cause of the seizure, the brain area affected the clinical manifestation and the associated medical condition which is grossly lacking in this current classification. Therefore, a different classification is suggested by Luders and colleagues, which take into cognizance the clinical semiology, location of the disease (epileptogenic zone), etiology, and comorbidity. This classification is called Four-Dimensional Epilepsy Classification System [29]. 

Four-dimensional Epilepsy Classification Systems: In this classification, a non-specific terminology "paroxysmal events‟ is used to refer to seizure-like events prior to a definitive diagnosis. When the diagnosis is made after having sufficient information based on the clinical presentation (such as clonic, tonic, tonic-clonic, myoclonic or autonomic), location of an event in the brain (like frontal, temporal, etc.), etiology (like a temporal tumor or hippocampal sclerosis) and comorbidity (like depression or anxiety), the paroxysmal events can then be categorized broadly into either epileptic paroxysmal events and non-epileptic paroxysmal events. The non-epileptic paroxysmal events are further divided into either psychogenic or organic paroxysmal events. For epileptic paroxysmal events, the following four dimensions must be considered: ictal semiology, epileptogenic zone, etiology, and comorbidities. For psychogenic non-epileptic paroxysmal events, the following three dimensions must be considered: semiology, etiology, and comorbidities. In this case, the semiology is defined by the same type of epileptic events, but the event is used in place of seizure. For organic non-epileptic events, three dimensions are also used: semiology, etiology, and comorbidities. However, semiology here is defined by non-epileptic, non-psychogenic events such as syncope, resting tremor, or cataplexy [29,30]. 

**Figure 4:**
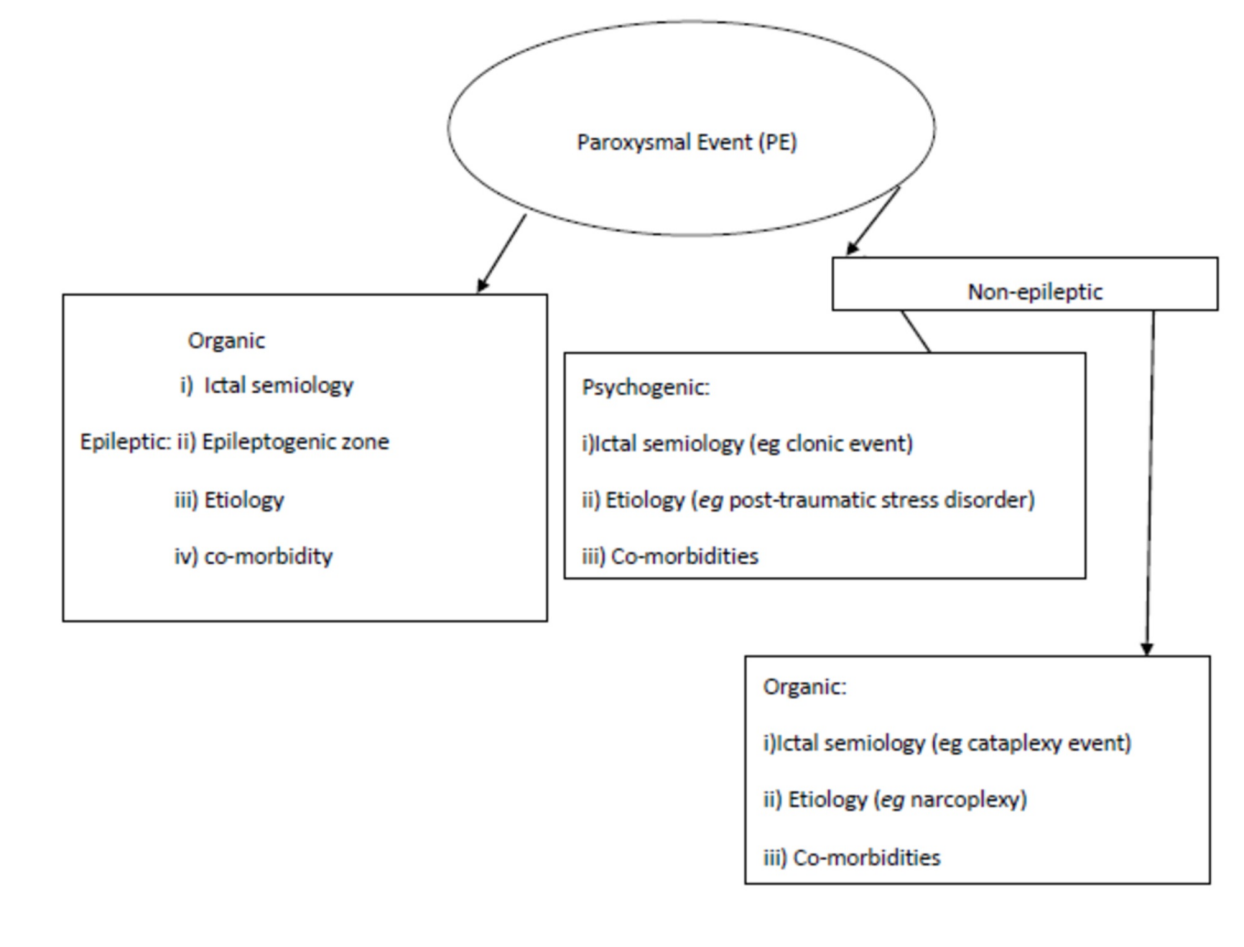
Four-dimensional Epilepsy Classification Systems

According to Loddenkemper et al., a five-dimensional classification should be adopted, which consists of the aforementioned four dimensions with seizure frequency as the fifth dimension [31]. Seizure frequency refers to the number of episodes of seizure events occurring over a given period of time. This has clinical significance as it indicates the severity of the disease. In line with this, the following terminologies could be defined: 1) Daily: when the seizure event occurs every day. 2) Persistent: when it occurs at least once in six months but not daily. 3) Rare: when it occurs at more than six months interval. 4) Undefined: when the frequency cannot be predicted. It includes breakthrough seizures that occur in a well-controlled patient due to either trigger like sleep deprivation or abrupt stoppage of medications [31]. Figure 4 below illustrate four-dimensional epilepsy classification system.

Definition of Terminologies: An epileptogenic zone is the part of the brain that is having abnormal electrical activities, and the clinical manifestations of these events are called epileptic seizures. The symptoms depend largely on the epileptogenic zone. The ictal semiology is the clinical symptom of epilepsy, such as clonic, tonic, atonic, or myoclonic. It helps in making a diagnosis and in starting appropriate antiepileptic drugs as it delineates whether the seizure is focal or generalized. Defining the seizure location is extremely important for proper management strategies, especially for surgical treatment. Defining etiology is also indispensable in the treatment and for prognostication. Identifying comorbidities will give a clear picture of the patient's problems, and many at times, it would be the main etiology of the patient's epilepsy and would greatly influence the management approach. For example, a patient with epilepsy and background renal or hepatic impairment will greatly improve if the comorbidity is properly managed. Also, antiepileptic drugs might differ in patients with different comorbidities [29,30]. 

Classification Based on Cortical Involvement

In this current classification, localization was limited only to either focal or generalized[11] without giving due consideration to the brain cortical localization. In terms of semiology, seizures arising from different parts of the cerebral cortex present with different clinical features. For example, frontal lobe seizure would differ considerably with temporal lobe seizure [29]. Additionally, classifying seizures and epilepsies based on cortical involvement has great therapeutic values, especially in presurgical evaluation, to determine with accuracy the location and extent of epileptogenic or seizure onset zone for surgical resection to produce seizure freedom [32]. Therefore, seizures and epilepsies should be classified based on the cortical part involved as either frontal, temporal, parietal, or occipital. Alternatively, it should be incorporated into the classification. 

Classification Based Exclusively on Clinical Semiology

Epilepsy is traditionally classified based on clinical features, EEG, and neuroimaging. However, in developing and resource-poor countries and in some primary care settings, both EEG and neuroimaging modalities may not be available, and physicians would have to rely solely on history and examinations. Therefore, ILAE should propose a classification of seizures and epilepsy in these settings based exclusively on clinical semiology[33]. Additionally, semiological classification even in the resource-rich setting would have the advantage of categorically differentiating between epileptic seizures and epilepsy syndromes in which the former would be diagnosed exclusively based on clinical features reported by the patient, direct observers or analysis of videos recorded during the seizure events, whereas the diagnosis of the latter would be based on the holistic analysis of the patient‟s condition including the clinical features, previous or family history of similar events, examination findings, laboratory test results and findings from EEG or neuroimaging[23]. We, therefore, propose the following semiological classification: 

Epileptic seizure: a) unilateral; b) bilateral (generalized); c) unilateral to bilateral.

Unilateral: 1) retained consciousness, and 2) impaired consciousness. Each can be motor or non-motor with all of their components.

Bilateral: 1) motor with all motor components 2) non-motor with all of its components.

Unilateral to bilateral: 1) motor, and 2) non-motor. 

Concept of "Seizure of Unknown Onset"

Seizure of unknown onset should be redefined. As it would affect the treatment of the patients as no specific drug is assigned for these forms of seizure and could turn out to be a severe seizure type after applying diagnostic investigations. This is particularly important in resource-poor settings. 

Comprehensive Instruction Manual

Lastly, we recommend that a new instruction manual be formulated. Which should contain detailed and clear criteria and guidelines on the role of comorbidities and etiological factors in classifying seizures and epilepsies. Additionally, further descriptors and identifiers regarding provoked and unprovoked seizures should be incorporated [8]. Additionally, emphasis should be made in the manual to consider the first level of epilepsy classification (seizure type) as clinical features of epilepsy, otherwise further classifying epilepsy would serve as a tautology. For instance, the focal seizure will automatically become focal epilepsy; thus, the former is completely unnecessary. However, it could be made to serve as a clinical presentation of focal epilepsy [13,29]. 

The ILAE mentioned that the classification could be used for both adults and children. However, some seizures occur almost exclusively in children like epileptic spasm (mainly in infants), while others occur mainly in older children and adults like generalized tonic-clonic, which is uncommon among infants. Therefore, we propose that an apparent dichotomy should be made between pediatric and adult epilepsies. 

## Conclusions

Attaining a standard classification for epilepsy has become a challenge for many years. Several models with different descriptors and terminologies that would allow the patients and healthcare givers understand their conditions were provided in each model, but not bereft of some significant limitations prompting subsequent review. This study extensively reviewed the ILAE classification of 2017 for seizures and epilepsy where limitations of the classification were thoroughly identified and recommendations were provided that should be considered during subsequent review of the classification model. We have categorically explained the terminologies used for seizures and epilepsy classification to help patients and their caregivers understand their conditions clearly for making an informed decision regarding their health conditions and for accurate reporting and description of their symptoms. Similarly, the extensive explanation of the terminologies provided in this review, will help healthcare givers make correct interpretations of patients' symptoms and then institute appropriate management. In this study we have suggested a four-dimensional classification model that could likely lead to improved understanding, prompt diagnosis and early management of epilepsy as it takes into cognizance the clinical semiology, disease location, etiology and associated comorbidities. This classification model would greatly help in better communication about epilepsy types among clinicians, the non-medical community, and researchers.
